# Knee instability caused by altered graft mechanical properties after anterior cruciate ligament reconstruction: the early onset of osteoarthritis?

**DOI:** 10.3389/fbioe.2023.1244954

**Published:** 2023-08-24

**Authors:** Janne Spierings, Marloes Van den Hengel, Rob P. A. Janssen, Bert Van Rietbergen, Keita Ito, Jasper Foolen

**Affiliations:** ^1^ Department of Biomedical Engineering, Orthopaedic Biomechanics, Eindhoven University of Technology, Eindhoven, Netherlands; ^2^ Institute of Complex Molecular Systems, Eindhoven University of Technology, Eindhoven, Netherlands; ^3^ Department of Orthopaedic Surgery and Trauma, Máxima Medical Centre Eindhoven/Veldhoven, Eindhoven, Netherlands; ^4^ Department of Paramedical Sciences, Health Innovations and Technology, Fontys University of Applied Sciences, Eindhoven, Netherlands

**Keywords:** anterior cruciate ligament reconstruction, graft remodeling, knee instability, osteoarthritis, knee biomechanics

## Abstract

Anterior cruciate ligament (ACL) rupture is a very common knee joint injury. Torn ACLs are currently reconstructed using tendon autografts. However, half of the patients develop osteoarthritis (OA) within 10 to 14 years postoperatively. Proposedly, this is caused by altered knee kine(ma)tics originating from changes in graft mechanical properties during the *in vivo* remodeling response. Therefore, the main aim was to use subject-specific finite element knee models and investigate the influence of decreasing graft stiffness and/or increasing graft laxity on knee kine(ma)tics and cartilage loading. In this research, 4 subject-specific knee geometries were used, and the material properties of the ACL were altered to either match currently used grafts or mimic *in vivo* graft remodeling, i.e., decreasing graft stiffness and/or increasing graft laxity. The results confirm that the *in vivo* graft remodeling process increases the knee range of motion, up to >300 percent, and relocates the cartilage contact pressures, up to 4.3 mm. The effect of remodeling-induced graft mechanical properties on knee stability exceeded that of graft mechanical properties at the time of surgery. This indicates that altered mechanical properties of ACL grafts, caused by *in vivo* remodeling, can initiate the early onset of osteoarthritis, as observed in many patients clinically.

## 1 Introduction

Anterior cruciate ligament (ACL) rupture is one of the most common knee joint injuries in young and active individuals (Zaid et al., 2015). The native ACL provides knee stability, by restraining anterior translation and internal rotation of the tibia relative to the femur (Perriman et al., 2018). To regain this joint stability after ACL rupture, the torn ACL is most often reconstructed using an autologous patellar tendon (PT) or semitendinosus tendon (ST), the latter occasionally in combination with a gracilis tendon (GT) graft (Janssen et al., 2017). However, one of the main clinical concerns after ACL reconstruction (ACLR) is the early onset of osteoarthritis (OA). In 31 percent of young adults, radiographic signs of OA are already observed 1 year postoperatively (Culvenor et al., 2015). Moreover, half of the patients develop symptomatic OA within 10 to 14 years after ACLR, resulting in the average age of patients who undergo total knee arthroplasty after previous ligament reconstruction to be 10 years younger than patients who did not have previous ligament reconstruction (Janssen et al., 2013; Cheung et al., 2020). An explanation for the high prevalence of OA after ACL reconstruction could be that ACL rupture was also accompanied by articular cartilage or meniscal damage. Alternatively, the mechanical properties of the graft could result in altered knee kine(ma)tics, which in turn results in OA. The latter defines the scope of this paper.

After ACLR, alterations in knee kinematics are found, indicating changed knee (in)stability. Knee instability is believed to be a contributor to the development and/or progression of OA (Dare and Rodeo, 2014; Blalock et al., 2015; Cheung et al., 2020; Kawabata et al., 2020). Previous research has shown that both anterior tibial translation (ATT) and tibial rotation are increased in ACL reconstructed knees compared to healthy contralateral controls (Scanlan et al., 2010; Struewer et al., 2012). Next to that, the altered tibial position in ACL reconstructed knees was shown to correlate with degenerative changes in the cartilage of the medial compartment (Zaid et al., 2015). Moreover, an increased ATT has been shown to significantly correlate with a higher degree of developed OA in patients in long-term follow-up (Struewer et al., 2012).

During ACLR surgery, the ruptured ACL is replaced with a PT, ST (/GT) graft. These grafts have a higher linear stiffness and a lower transition strain (the strain where the recruitment of crimped collagen fibers starts/the end of the toe region) than the native ACL (West and Harner, 2005; Chandrashekar et al., 2008). After implantation, these grafts undergo a remodeling response in which the stiffness and strength of the graft decrease until around 6–8 weeks postoperatively and can drop to 10% of the native ACL (Janssen and Scheffler, 2014). Subsequently, mechanical properties of the grafts recover over time, but only to 50%–60% of the native ACL (Janssen and Scheffler, 2014). Moreover, the grafts lengthen over time, resulting in increased graft laxity (Chandrashekar et al., 2008). It is expected that these changes contribute to knee instability and, therefore, the development and/or progression of OA by altering the type and magnitude of loading that is applied to the articular cartilage.

Based on the results of previous *in vivo* studies using minipigs that underwent ACLR, there is presumed to be a negative correlation between graft stiffness and the area and severity of cartilage damage (Kiapour et al., 2017; Beveridge et al., 2019). Likewise, previous research using Finite Element (FE) modeling has shown that the graft stiffness influences ATT, e.g., lower graft stiffness results in higher ATT (Suggs et al., 2003; Peña et al., 2005), and cartilage peak pressure, e.g., lower graft stiffness results in increased cartilage pressure (Li et al., 2002; Smith et al., 2015). Moreover, the stiffness of the ACL was found to correlate to the location of the center of the cartilage pressure (Smith et al., 2015). It was also shown that the positioning and tensioning of ACL grafts are important to recover joint kinematics and kinetics and that the higher stiffnesses of the tendon grafts at implantation result in increased tibial cartilage stress and strain (Halonen et al., 2016; Naghibi et al., 2020). Next to that, Halonen and coworkers suggest that both the stiffness of the graft and the pre-strain applied to the graft during surgery affect knee motion (translational and rotational motions) (Halonen et al., 2016). However, current studies do not include the change in graft mechanical properties due to graft remodeling. This is important as it has been suggested that the decrease in graft stiffness and increase in graft laxity in particular contribute to the (in)stability of the knee joint. Therefore, this research aims to assess changes in knee kine(ma)tics and cartilage loading, as a measure of knee (in)stability during graft remodeling. This will be analyzed using subject-specific FE knee models and adjusting the graft material behavior to obtain a decreased graft stiffness and/or increased transition strain.

## 2 Material and methods

In this research, 4 subject-specific knee geometries were obtained from the second-generation Open Knee models (Bennetts et al., 2015; Bonner et al., 2015; Colbrunn et al., 2015; Erdemir et al., 2015; SimKT, 2015). These models contain the femur, tibia, patella, and fibula with their corresponding cartilage layers, the menisci, the four major knee ligaments (ACL, PCL, MCL, and LCL), and the quadriceps and patellar tendon. The models were selected based on the criteria that no, radiographically visible, tibial cartilage damage was present (Chokhandre et al., 2021). Subject details can be found in Table 1. The geometries were uploaded into the FEBio Software (Maas et al., 2012).

**TABLE 1:** Subject details of the corresponding Open Knee models (obtained from (Open knee(s): virtual biomechanical representations of the knee joint, 2015)).

Subject	1: OKs001	2: OKs002	3: OKs003	4: OKs008
Knee (left/right)	Right	Right	Left	Right
Gender (male/female)	Male	Female	Female	Male
Age (years)	71	67	25	40
Height (m)	1.83	1.55	1.73	1.78
Weight (kg)	77.1	45.3	68.0	63.5
BMI	23.1	18.9	22.8	20.1

**FIGURE 1:**
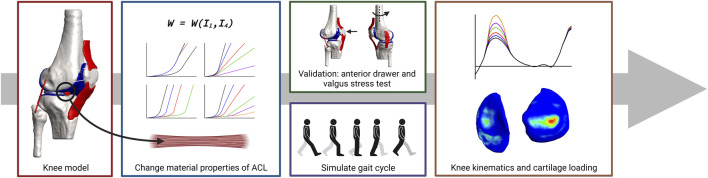
Schematic overview of the workflow used in this study. Subject-specific knee geometries were obtained from the Open Knee project. The material behavior of the native ACL was changed by changing the material properties of the constitutive law of the ACL to create tendon grafts or grafts with a decreasing stiffness and/or increasing transition strain to mimic graft remodeling. Validation was done by simulating an anterior drawer and a valgus stress test. The influence of graft remodeling was evaluated by simulating a gait cycle and recording the knee kinematics and tibial cartilage loading.

For the creation of the models used in this study, the suggestions reported by the Open Knee project were implemented and summarized below. This includes the discretization of the geometries, the material properties of the original objects, and the contact definitions. The creation of the grafts mimicking graft remodeling and the addition of pre-strain to the tendons and ligaments is novel. Figure 1 shows a schematic overview of the workflow used in this study. To note, in this research, stability is referred to as unchanged kine(ma)tics while changing mechanical properties of the ACL.

### 2.1 Finite element model

The subject-specific knee geometries were discretized using four-node tetrahedral elements (TET4) with one integration point for all soft tissues and, to save computational time, three-node triangular shell elements (TRI3) for all bones.

The material behavior of all tissues was described using constitutive models that provide the material properties of the tissues. Bones are defined as rigid bodies since the elastic modulus of bone is orders of magnitude larger than that of soft tissue (Peña et al., 2006; SimKT, 2015; Benos et al., 2020). Cartilage was modeled as a nearly incompressible Neo-Hookean material (Haut Donahue et al., 2002; SimKT, 2015). The menisci were described as nearly incompressible, transversely isotropic, hyperelastic with reinforced fibers and a Neo-Hookean ground substance (Chokhandre et al., 2023). The fiber orientation was obtained by fitting a circle through each meniscus and aligning the fibers in the tangential direction (Shriram et al., 2017). Ligaments, tendons, and grafts were modeled as uncoupled nearly incompressible, transversely isotropic, hyperelastic materials with a Neo-Hookean ground substance. This material behavior accommodates tensile dominant behavior of the collagen fibers and neglects time-dependent behavior, such as viscoelasticity, justified by the high ratio between the viscoelastic time constant of the material and the short loading time of interest (Wu and Ladin, 1996; Hirokawa and Tsuruno, 2000; Weiss and Gardiner, 2001; Weiss et al., 2005; Peña et al., 2006). The deviatoric part was determined by the isotropic matrix and the one directional resistance of the fibers. The volumetric part was determined by the volume change. The strain energy density function $W=W\;\left(I_{1},I_{4}\right)$ was given by:

$$W=F_{iso}\left({\tilde{I}}_{1}\right)+F_{fiber}\left({\tilde{I}}_{4}\right)+\frac{\kappa }{2}{\left[\ln\left(J\right)\right]}^{2}$$

Where $F_{iso}=C_{1}\left({\tilde{I}}_{1}-3\right)$ , which represents the contribution of the isotropic matrix with $C_{1}$ a constant, ${\tilde{I}}_{1}$ the 1^st^ invariant of the deviatoric right Cauchy Green deformation tensor $\tilde{C}={\tilde{F}}^{T}∙\tilde{F}$ with $\tilde{F}=J^{-\frac{1}{3}}F$ , where F is the deformation gradient, J the Jacobian of the deformation given by J=det⁡F . $F_{fiber}$ represents the contribution of the fibers with ${\tilde{I}}_{4}=a_{0}∙\tilde{C}∙a_{0}={\tilde{\lambda }}^{2}$ where $a_{0}$ represents the initial fiber direction and $\tilde{\lambda }$ the stretch in the fiber direction. The bulk behavior was represented by $\frac{\kappa }{2}{\left[\ln\left(J\right)\right]}^{2}$ , with κ the bulk modulus, which is the volumetric part.

The fiber contribution was described with the following equations:

$$\left\{\begin{array}{c} \tilde{\lambda }\frac{\partial F_{fiber}}{\partial \tilde{\lambda }}=0\;for\;\tilde{\lambda }\leq 1\\ \tilde{\lambda }\frac{\partial F_{fiber}}{\partial \tilde{\lambda }}=C_{3}\left(e^{C_{4}\left(\tilde{\lambda }-1\right)}-1\right)\;for\;1<\tilde{\lambda }\leq \lambda_{m}\\ \tilde{\lambda }\frac{\partial F_{fiber}}{\partial \tilde{\lambda }}=C_{5}+C_{6}\tilde{\lambda }\;for\;\tilde{\lambda }\geq \lambda_{m} \end{array}\right.$$

Where $C_{3}$ is the exponential stress coefficient, $C_{4}$ the uncrimping fiber coefficient, $C_{5}$ the modulus of the straightened fibers, and $\lambda_{m}$ the stretch by which the fibers are straightened. $C_{6}$ can be calculated for continuous stress at $\lambda_{m}$ using $C_{6}=\frac{1}{\lambda_{m}}\left[C_{3}\left(e^{C_{4}\left(\lambda_{m}-1\right)}-1\right)-C_{5}\right]$ . All material properties were based on previous research (Peña et al., 2005; Shriram et al., 2017). An overview can be found in Supplementary Table S1.

Since ligaments experience *in situ* strain in the body, a pre-strain was prescribed to the reference configuration according to the method developed by Maas *et al.* (Maas et al., 2016). The values for *in situ* stretch ( $\lambda_{p}$ ) were based on the average of experimental fiber stretch data (Gardiner et al., 2001; Peña et al., 2006; Dhaher et al., 2010) (Supplementary Table S1).

**FIGURE 2:**
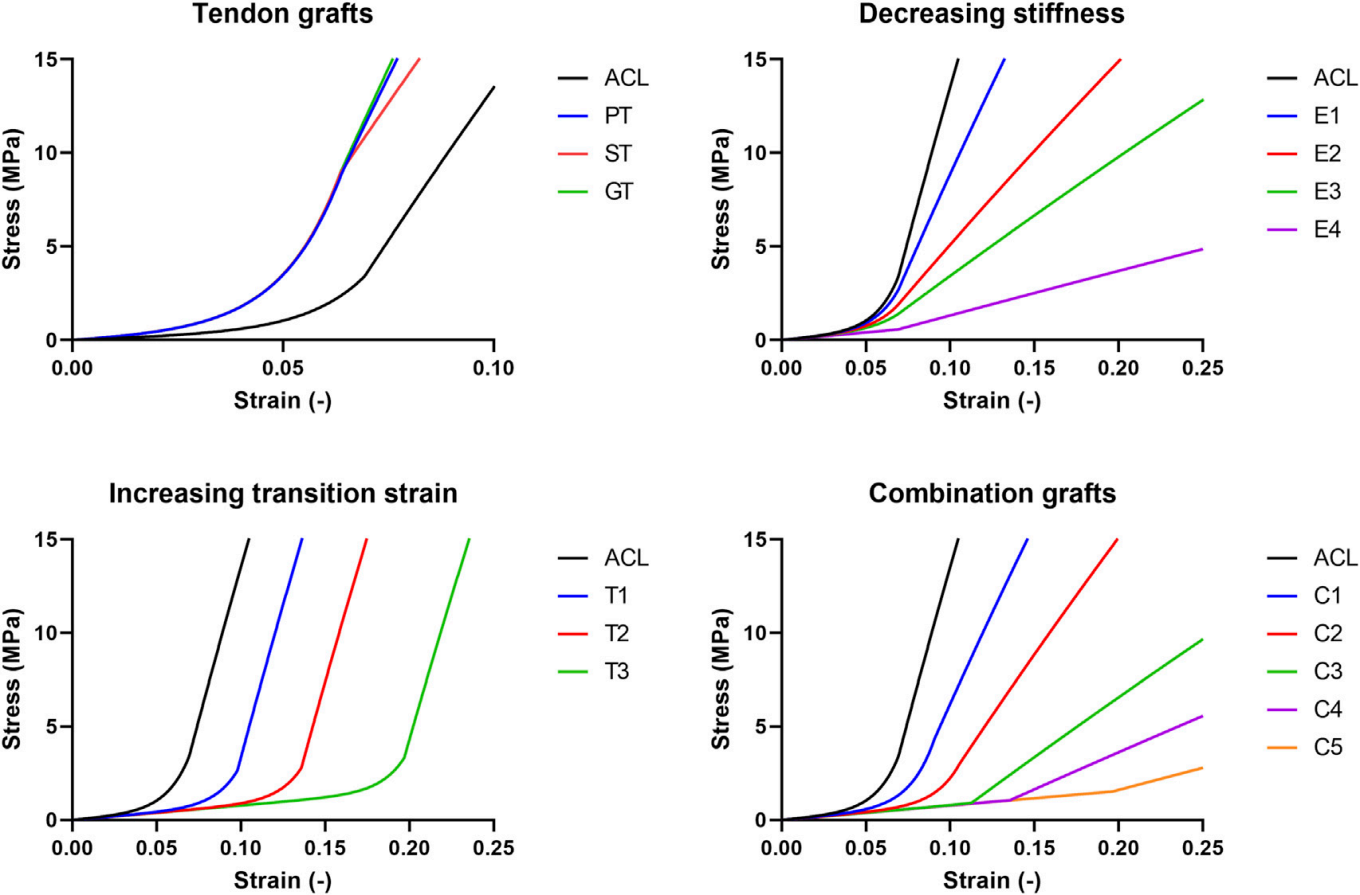
Schematic overview of the stress-strain relations of the grafts created and used in this research.

Next, to examine the influence of postoperative alterations in graft mechanical properties, as a result of graft remodeling, (hypothetical) grafts were created. To note, in this research the focus was on the effects of graft remodeling after ACLR by altering stiffness and length of the toe region of the existing ACL in the models, excluding the effects of ACL attachment location and changes in morphology or structure. Since graft stiffness and transition strain are not direct parameters of the Neo-Hookean material model, graft remodeling was modeled by tuning the Neo-Hookean constants ( $C_{1},C_{3},C_{4},C_{5}$ ) to result in smooth stress-strain behavior, in combination with values for stiffness and transition strain that are clinically relevant. Stiffness values are based on the commonly believed physiological values of 20% after the proliferation phase and 60% after the ligamentization phase of graft remodeling (Janssen and Scheffler, 2014). The values for the transition strain were based on side-to-side differences in knee stability postoperatively (Fleming et al., 2002; Kim et al., 2006; Nakanishi et al., 2020; Michel et al., 2022). Figure 2 gives a schematic overview of the stress-strain relations of the created grafts.

To mimic a decreased graft stiffness (68%, 37%, 24%, and 9% of the native ACL, further referred to as E1 to E4 respectively), Neo-Hookean constant $C_{5}$ was decreased as this represents the modulus of the straightened fibers. For a smooth transition from the heel to the linear region, the fiber uncrimping coefficient, $C_{4}$ , was decreased as well. To mimic an increased graft laxity, grafts with an increased transition strain (143%, 200%, and 286% of the native ACL, further referred to as T1 to T3 respectively) were created by decreasing the exponential stress coefficient $C_{3}$ , as this will elongate the toe region of the stress-strain curve. Next to that, the stretch at which the fibers straighten, $\lambda_{m}$ , was increased to shift the transition from the non-linear to the linear region of the stress-strain curve towards a higher strain. Moreover, the decreased stiffness and increased transition strain were combined to create combination grafts, where the stiffness decreases to 68%, 47%, 24%, 15%, and 9% respectively, and the transition strain increases to 107%, 136%, 171%, 250%, and 286% respectively, further referred to as C1 to C5. The patellar tendon (PT; 133% stiffness, 79% transition strain), the semitendinosus tendon (ST; 107% stiffness, 79% transition strain), and the gracilis tendon (GT; 147% stiffness, 79% transition strain) were used to mimic the mechanical behavior and mechanical properties of the graft after implantation before the onset of graft remodeling (Peña et al., 2005). Supplementary Table S1 gives an overview of the material properties of the created grafts.

For analysis, these grafts were implemented in the Open Knee models and the stance phase of the gait cycle was analyzed. It was assumed all (hypothetical) grafts, including the PT, ST, and GT, experienced an *in situ* stretch of 1.016, similar to the native ACL (Supplementary Table S1).

The contact between rigid bodies and soft tissues was assumed to be rigid, and all contacts between two soft tissues were modeled as a sliding elastic contact to enable frictionless sliding and to prevent penetration (Risvas et al., 2022). For the contacts between the tibial and femoral cartilage and the menisci, the femoral cartilage and menisci were assigned as primary surfaces with the tibial cartilage acting as secondary surface. In this way, contact pressures of both the femoral cartilage and the menisci are visible on the tibial cartilage.

### 2.2 Knee motion

**FIGURE 3:**
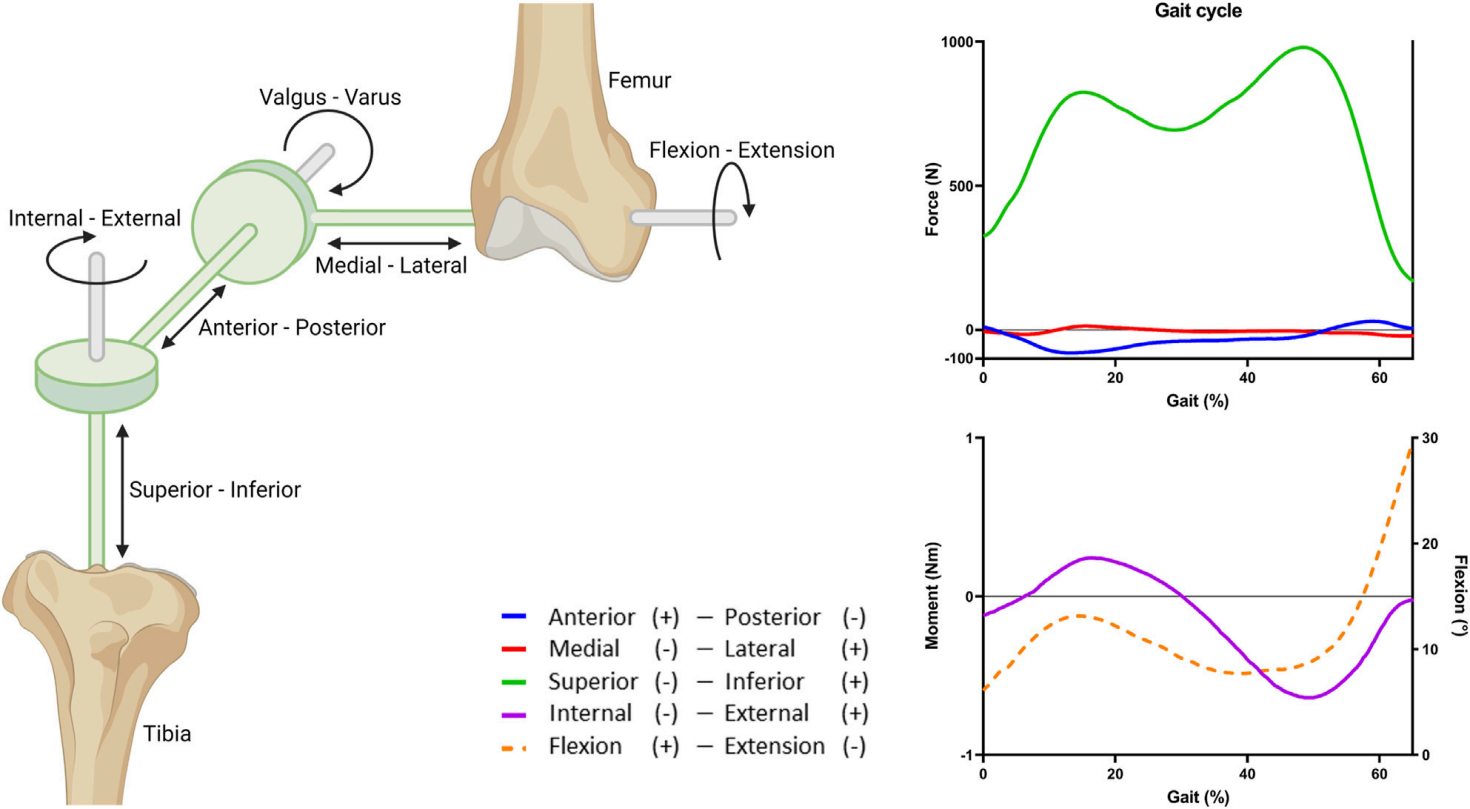
Overview of the joint coordinate system and the applied gait cycle loads. Three rigid cylindrical joints are connected through a four-link kinematic chain to create 6 DoFs. The imaginary links and rigid bodies are shown in green. The stance phase of the gait cycle was simulated by applying fractions of the forces, moment, and rotation obtained from the OrthoLoads database (Bergmann et al., 2014). Flexion (dashed line) is portrayed on the right *y*-axis.

Knee motion was described using 6 degrees of freedom (DoF), three translational and three rotational movements using a four-link kinematic chain with three cylindrical joints, as already implemented by the Open Knee project (Grood and Suntay, 1983). In short, this was implemented using two imaginary links defined by two imaginary rigid bodies, used to create the three cylindrical joints (Figure 3). A cylindrical joint is a rigid connector that connects two rigid bodies and exerts a reaction force or moment on the rigid body at the origin of the connector. The rigid cylindrical joint defines two DoFs of the bones relative to each other: one translational and one rotational, along the joint axis of the rigid connector. The joint axes were determined by anatomical landmarks obtained from the 3D geometries of the subjects (Erdemir, 2015). Along the epicondylar femoral axis, medial-lateral translation and flexion-extension were applied. Along the tibial long axis, superior-inferior translation and internal-external rotation were applied. Next, along the axis perpendicular to both the femoral and tibial long axis, anterior-posterior translation and valgus-varus rotation were applied (Figure 3).

### 2.3 Gait cycle

The tibial cartilage loading during knee motion was studied by simulating the stance phase of a gait cycle (defined as 65% gait). The stance phase was simulated by applying a force, moment, and rotation to the femur (Figure 3). The tibia and fibula were fixed in all DoFs. The applied loads were based on average *in vivo* loads, assuming a body weight of 75 kg, obtained from the OrthoLoads database (Bergmann et al., 2014). For simplicity and because of the lack of information on alignment of the knee joint relative to the vertical axis, valgus and varus moments were excluded (Naghibi et al., 2020).

Because of the non-linear behavior of the tissues, the loads were applied in steps, first, the pre-strain was applied, then the initial values for force, moment, and rotation of the stance phase, and finally the gait loads during the stance phase. These loads were assigned through the rigid connectors. To obtain translation and rotations similar to the *in vivo* findings by Gray *et al.* (Gray et al., 2019), only a fraction of the loads was applied (50% force, 10% moment, and 60% flexion; Supplementary Figure S1). The comparison to the findings by Gray *et al.* was done to select a set of input parameters that corroborate with knee kinematics that were in range of *in vivo* data, as reported by Gray *et al.* The main reason for this approach is that the model does not contain stabilizing factors like muscles and muscle action.

### 2.4 Verification

#### 2.4.1 Valgus stress test

To verify the effect of the pre-strain, a valgus stress test was simulated both with and without ( $\lambda_{p}$ = 1.0) applied pre-strain in the MCL, as an arbitrary example (Supplementary Table S1). A valgus stress test is used to assess the integrity of the MCL (Aronson et al., 2010). Valgus was simulated by applying a torque of 10 Nm to the origin of the adduction-abduction rigid cylindrical joint while keeping the other DoFs free. Both the tibia and fibula were fixed in all DoFs. By comparing the pre-strain stretch in the MCL, the contact force on the lateral condyle, and the valgus torque over valgus rotation, with and without applied *in situ* stretch, the influence of the applied pre-strain could be investigated. It was assumed that the results of the valgus stress test were representative of the other tendons and ligaments.

#### 2.4.2 Anterior drawer test

To verify whether the created changes in graft mechanical properties indeed resulted in increased knee range of motion, an anterior drawer test was simulated. In clinical settings, an anterior drawer test is performed to determine knee (in)stability (Zhao et al., 2021). This test was simulated by applying an anterior force of 134 N on the femur through the origin of the adduction-abduction rigid connector, which is in essence the same as applying an anterior tibial load (Fu et al., 1999; Yagi et al., 2002; Peña et al., 2006). The fibula and tibia were fixed in all DoFs. The knee was kept in full extension by fixing the flexion angle at zero degrees. Outcomes of these simulations were compared to experimental data found in literature (Höher et al., 2001; Yagi et al., 2002; Zantop et al., 2007; Kim et al., 2015; Drews et al., 2017).

### 2.5 Quantification of knee range of motion, maximum tibial cartilage contact pressure, and the location of the contact pressure

Knee motion of the rigid connectors and tibial cartilage contact pressure on all nodes of the tibial cartilage were recorded during the stance phase of gait. Next to that, the location of the tibial cartilage contact pressure was quantified using the weighted center of mass on grayscale images of the contact pressure on the tibial cartilage objects in ImageJ (Schneider et al., 2012). Using the coordinates of the weighted center of mass and the dimensions of the tibial cartilage objects, the shift in the medial (−)—lateral (+) and anterior (+)—posterior (−) direction was quantified.

## 3 Results

### 3.1 Graft mechanical properties influence knee range of motion

**FIGURE 4:**
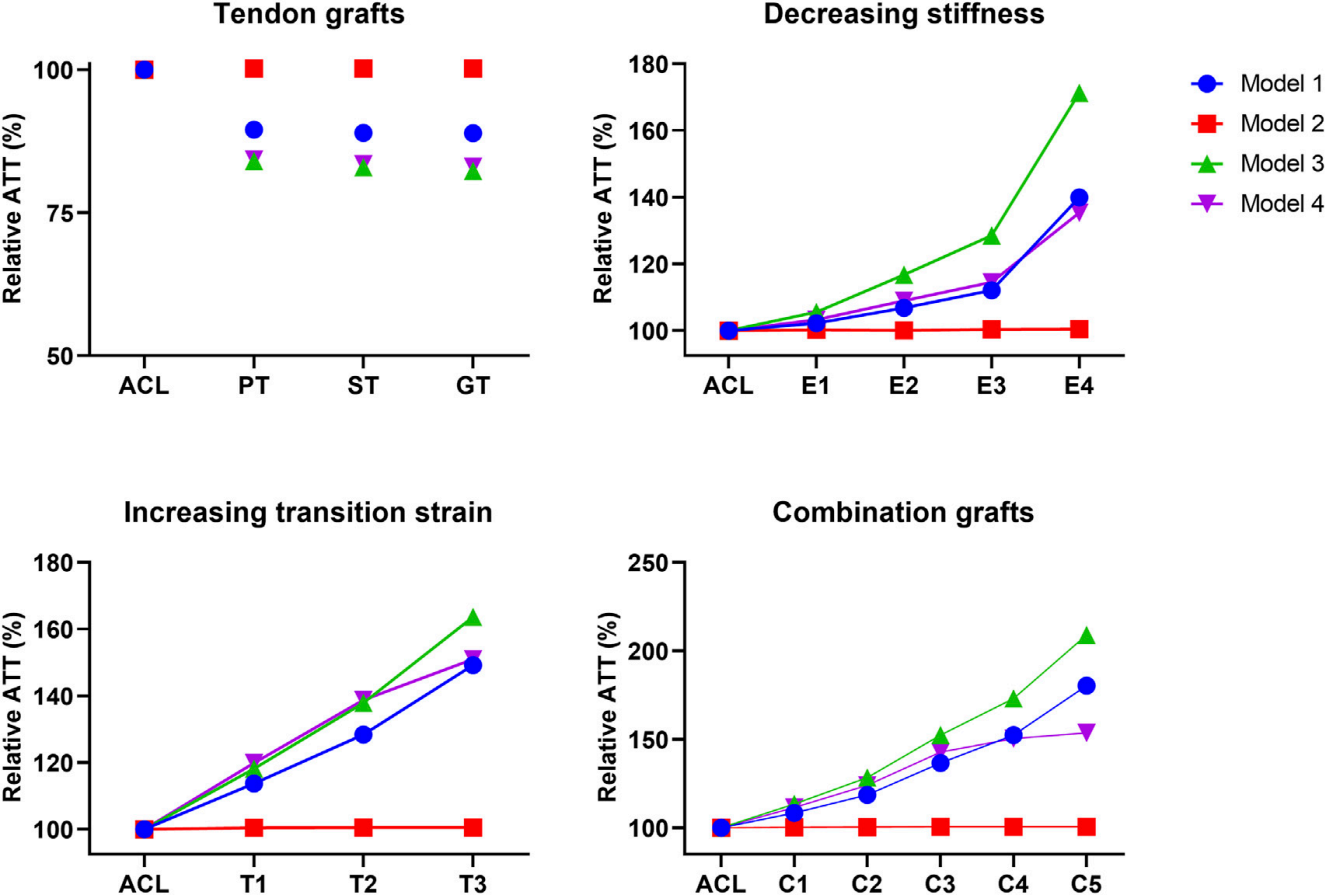
Altering graft mechanical properties results in changed anterior tibial translation. Difference in anterior tibial translation (ATT) for tendon autografts, grafts with a decreasing stiffness, grafts with an increasing transition strain, and a combination of both.

**FIGURE 5:**
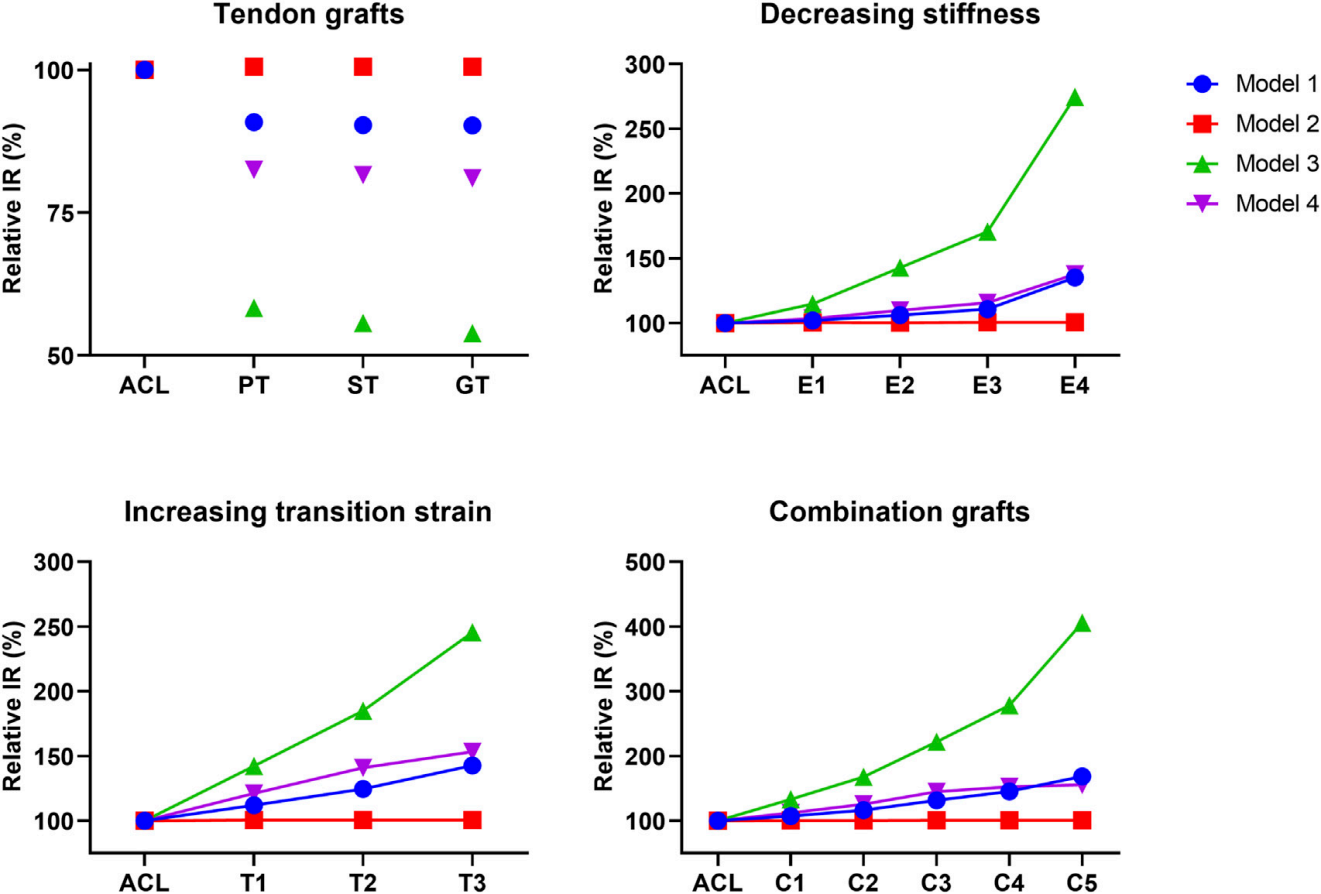
Altering graft mechanical properties results in changed internal tibial rotation. Difference in internal tibial rotation (IR) for tendon autografts, grafts with a decreasing stiffness, grafts with an increasing transition strain, and a combination of both.

The effect of graft remodeling on the anterior tibial translation and internal tibial rotation (IR) was evaluated. Results were analyzed at the point of maximum force on the ACL during the stance phase (Bergmann et al., 2014; Belbasis et al., 2015; Gray et al., 2019). For the ATT, it can be observed that there is a small decrease (10%–17%) for the PT, ST, and GT grafts in 3 of the 4 models (Figure 4). For grafts with a decreasing stiffness, an exponential increase in ATT in 3 of the 4 models was found. Grafts with an increasing transition strain showed a more linear increase in ATT, again in 3 out of the 4 models. When both the graft’s stiffness decreased and the transition strain of the graft increased, the increase in ATT surpassed the increase from the individual effects (Figure 4). For the IR, similar situations were observed, with an even larger increase found in model 3 (Figure 5). Again, there was an exponential increase found with a decreasing graft stiffness. For an increased graft laxity, the increase in IR was found to be more linear. A decreased stiffness combined with an increased graft laxity exceeded the individual effects found (Figure 5). Together, these results indicate that the altered graft mechanical properties due to *in vivo* graft remodeling affect knee kinematics by increasing anterior tibial translation and internal rotation of the knee.

### 3.2 Graft mechanical properties influence the tibial cartilage contact pressure during gait

Tibial cartilage loading during graft remodeling was evaluated. Tibial cartilage loading was visualized and evaluated at the point of maximum force on the ACL during the stance phase. There were no significant differences found in the maximum contact pressure between the different grafts with respect to the native ACL (one-way ANOVA, *p* > 0.99, Supplementary Figure S4).

**FIGURE 6:**
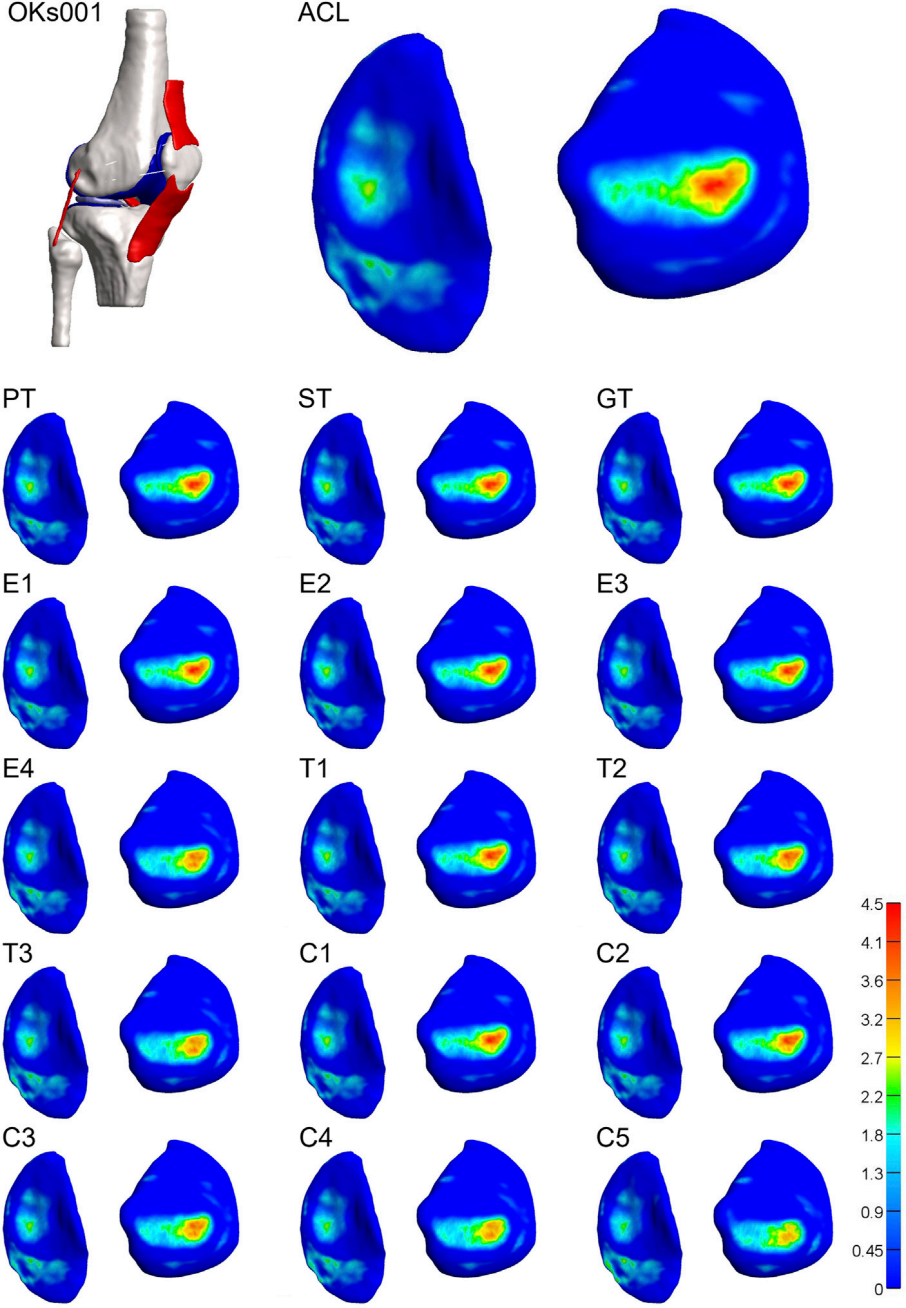
Altering graft mechanical properties results in a relocation of tibial cartilage contact pressure [MPa]. Visual representation of the tibial cartilage contact pressure distribution of model 1 for the native ACL, the tendon grafts (PT. ST, and GT), grafts with a decreasing stiffness (E1-4), grafts with an increasing transition strain (T1-3), and a combination of both (C1-5). Left: medial tibial cartilage; right: lateral tibial cartilage.

**FIGURE 7:**
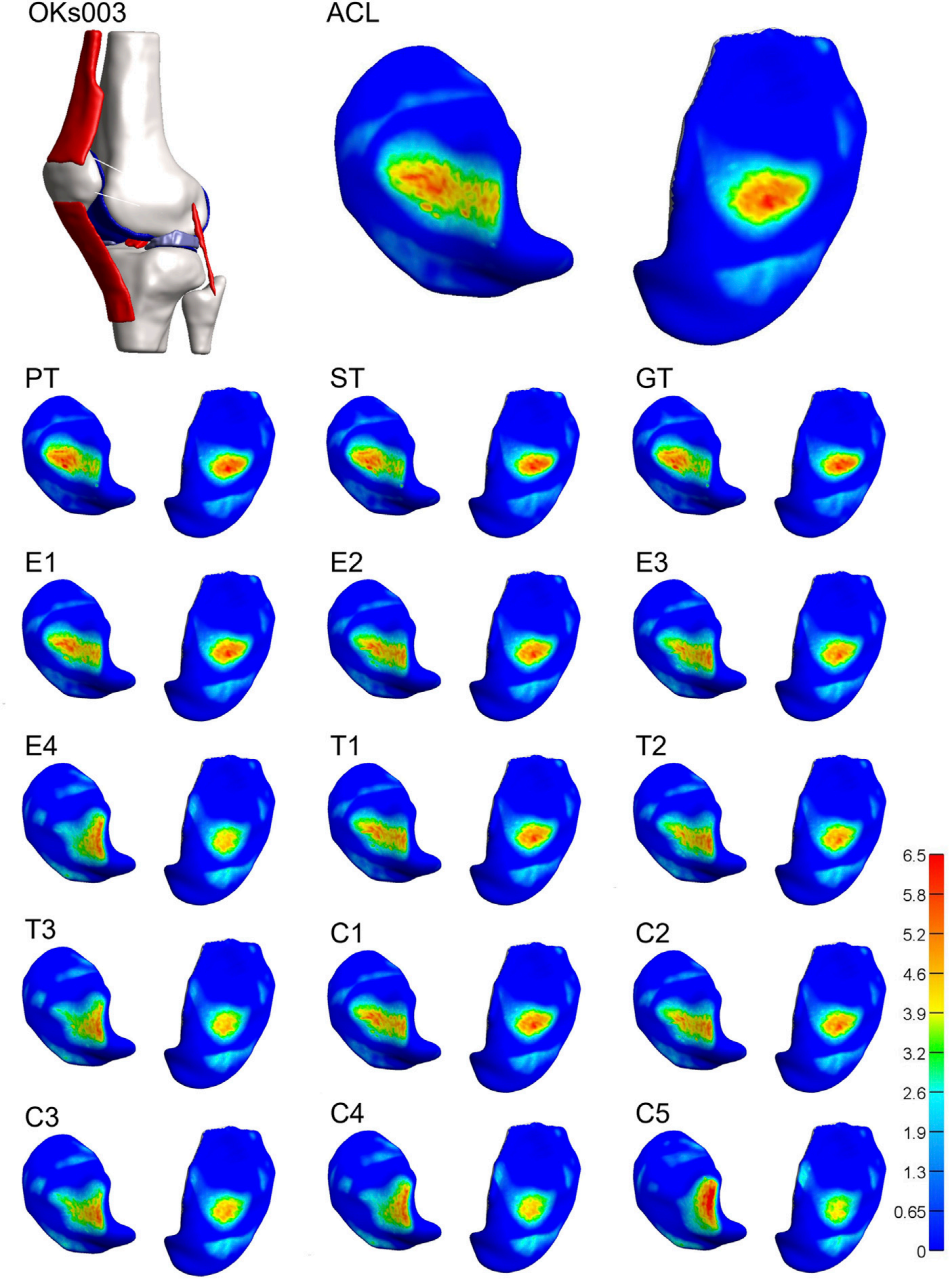
Altering graft mechanical properties results in a relocation of tibial cartilage contact pressure [MPa]. Visual representation of the tibial cartilage contact pressure distribution of model 3 for the native ACL, the tendon grafts (PT, ST, and GT), grafts with a decreasing stiffness (E1-4), grafts with an increasing transition strain (T1-3), and a combination of both (C1-5). Left: lateral tibial cartilage; right: medial tibial cartilage.

**FIGURE 8:**
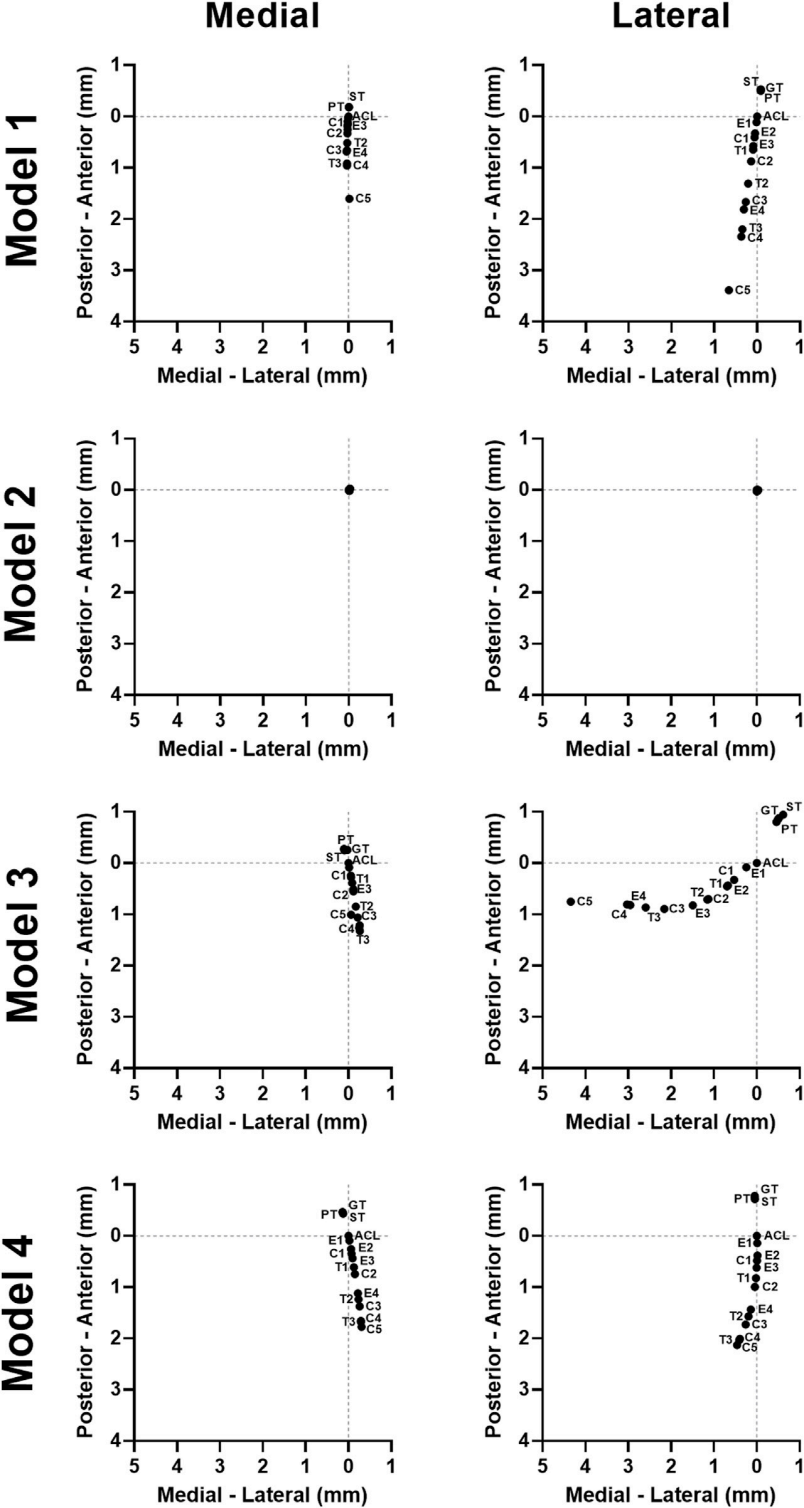
Graft mechanical properties influence the location of the tibial cartilage contact pressure in a subject-specific direction. The quantified weighted center of mass of the contact pressure on the tibial cartilage for all models.

For 3 of the 4 models, both stiffness and transition strain influenced the location of the contact pressure (weighted center of mass) (Figure 6, Figure 7; Supplementary Figures S5, S6, Supplementary Videos S1–S4). It was found that the location of the contact pressure for the grafts with a higher linear stiffness and a lower transition strain (PT, ST, and GT) only differed by a maximum of 1 mm compared to that of the native ACL. The direction of the relocation was found to tend towards a location more anterior and distant from the center of the knee (Figure 8; Supplementary Video S1). The lower the stiffness of the graft, the greater the relocation of the contact pressure tended towards a location more posterior and closer to the center of the knee. This was especially the case for the lateral tibial cartilage, with major relocations (>1 mm) found for stiffnesses <15% of the native ACL (Supplementary Video S2). Similar results were found for an increasing transition strain, with relocation >1 mm found for transition strains twice or more than that of the native ACL (Supplementary Video S3). Interestingly, both these relocations were found to be in the opposite direction than the tendon grafts, which are stiffer with a lower transition strain (Figure 8). Overall, the combination grafts followed the same pattern, with a more extreme shift in contact pattern found. More specifically, for subject 1 the relocations were greater for the combined effects than for the individual effects alone, with the shift corresponding to C5 (9% stiffness and 286% transition strain) nearly being the sum of the individual effects of E4 (9% stiffness) and T3 (286% transition strain) for both the medial and lateral cartilage (Figure 6, Figure 8). On the other hand, in subject 4 the relocation of the combination grafts was found to be far from a synergistic effect. For the lateral cartilage, the relocation corresponding to C2 (47% stiffness and 136% transition strain) was smaller and in the opposite direction than the shift corresponding to E2 (37% stiffness), indicating that the transition strain influenced the location of the peak pressure to a greater extent (Figure 8; Supplementary Figure S6). In subject 3, the combination grafts resulted in a greater relocation of the contact pressure than for the individual effects as well. Although, for C5 the shift in the anterior-posterior direction was smaller than that of the individual effects for the lateral cartilage. This is the result of higher peak pressures in other locations than the main peak pressure (Figure 7, Figure 8). Taken together, the location of the cartilage peak pressure depends on the stiffness and transition strain of the graft and the subject-specific knee geometry.

### 3.3 Verification: valgus stress and anterior drawer test

A valgus test was simulated to verify whether the pre-strain was applied correctly. The pre-strain stretch in the MCL was visualized for a valgus rotation of 3° in a situation where pre-strain was applied and one in which no pre-strain was applied (Supplementary Figure S2). In the situation where no pre-strain was applied, the pre-strain stretch is a result of solely the valgus rotation, whilst it is the sum of the applied pre-strain and valgus rotation in the situation where pre-strain was applied. As can be seen in Supplementary Figure S2, for all four models the pre-strain stretch in the MCL is higher when pre-strain was applied to the ligaments. Next to that, for the same degree of valgus rotation, higher valgus torque and contact force in the lateral condyle were found. Thus, the addition of pre-strain in the ligaments resulted in stiffer knee rotations.

Besides, as one of the functions of the ACL is to restrain ATT, grafts with decreased mechanical properties, theoretically, should result in an increased ATT. To verify this, an anterior drawer test was simulated, and the resulting ATT was compared to experimental values obtained from literature. Supplementary Figure S3 shows the ATT for the native ACL and the created grafts for all four models. The ATT values of the native ACL were found to be within the range found in literature. For grafts with a decreased stiffness, the ATT seems to follow an exponential trend, while for grafts with an increased transition strain, the increase in ATT was found to be more linear. For ACL deficiency (DEF) the ATT values obtained from the models were higher than the values found in literature, which might be explained by possible active muscle restriction of the patient themselves in a clinical setting. Nevertheless, decreasing graft stiffness and increasing graft laxity, result in an increased ATT, as expected.

## 4 Discussion

One of the main clinical concerns after ACLR is the early onset of OA which occurs in approximately 50% of the, mostly adolescent, patients (Cheung et al., 2020). It is known that the higher stiffnesses of tendon grafts at surgery result in increased tibial cartilage stresses and strains (Halonen et al., 2016; Naghibi et al., 2020). However, recent studies did not consider the remodeling of these grafts. During this remodeling, the stiffness of the graft decreases while the graft laxity increases (Chandrashekar et al., 2008; Janssen and Scheffler, 2014). Therefore, this study aimed to investigate changes in knee kine(ma)tics and tibial cartilage loading during ACL graft remodeling. The main findings of this research are that a decreasing graft stiffness and/or an increasing graft laxity increase(s) both the ATT and IR and relocates the center of the cartilage pressure. It was demonstrated that the PT, ST, and GT grafts (mechanical properties at the time of surgery) had a smaller influence on knee kine(ma)tics than the altering mechanical properties caused by the *in vivo* remodeling process of those grafts. A decreasing graft stiffness and/or an increasing graft laxity increased ATT and IR, both movements the native ACL restricts, indicating altered knee kinematics. Together with a relocation of the tibial cartilage contact pattern, which suggests abnormal loading of the knee, it is clear that graft remodeling after ACLR can result in knee instability. Important to note, these changes were not observed in all models, indicating that this may be a subject-specific risk, in analogy with clinical outcome where not all ACLR patients develop OA. We propose that this instability can be an initiator for the development of OA for a subset of patients.

In this research, the difference found in knee range of motion or cartilage loading when replacing the ACL with a PT, ST, or GT graft (mechanical properties at the time of surgery) were relatively small, which is in line with previous research. Halonen *et al.* (Halonen et al., 2016) and Nagibi *et* al. (Naghibi et al., 2020) found a minor increase in cartilage strain or contact pressure and a minor decrease in knee range of motion (values not mentioned) when using tendon grafts in ACLR. Next to the fact that these changes are only minor, a few weeks after ACLR, the graft mechanical properties have already altered due to graft remodeling. Therefore, regardless of whether the peak pressures on the cartilage may be slightly increased, it is less likely to strongly relate to the development of OA, as this loading configuration only lasts for a maximum of a few weeks.

For grafts with a decreasing stiffness, the ATT shows an exponential increase, with high values in particular for stiffnesses <24% (E3). Similar results are reported in a study by Li *et al.* (Li et al., 2002), who modeled partial ACL ruptures by decreasing the stiffness of the ACL and found a major increase in ATT for a graft stiffness of 25% compared to the native ACL. On the other hand, Chandrashekar *et al.* (Chandrashekar et al., 2008) suggested that the linear stiffness of grafts only has a minor influence on the biomechanics of the knee joint compared to the mechanical properties of the low-load region. In this study, the ATT and IR increased linearly with an increasing graft laxity, which shows that the mechanical properties in the low-load region indeed influence knee kinematics as well. Likewise, in this study, it was found that the transition strain had a greater influence on the location of the tibial cartilage peak pressure than the linear stiffness of the graft. This points to the importance of applying the correct graft tension at surgery, as a too-low or a too-high pre-tension results in increased knee instability (Halonen et al., 2016).

Next to an increased knee range of motion, a relocation in cartilage contact pressure was found with altered graft mechanical properties. Similar to the study by Smith *et al.*, (Smith et al., 2015), it was found that a decrease in graft stiffness relocated the peak pressure more posterior on the tibial plateaus. In this study, this relocation was found to be more evident on the lateral cartilage, while radiographically often the first cartilage damage after ACLR is found on the medial cartilage which is also more commonly affected than the lateral cartilage (Cheung et al., 2020), which can be explained by a higher prevalence of cartilage damage on the medial side already present at the time of surgery (Everhart et al., 2020). Still, the change in contact pressure pattern indicates abnormal cartilage loading during gait, which is believed to influence OA development (Blalock et al., 2015; Kawabata et al., 2020; Naghibi et al., 2020), e.g., because the peak pressure relocates to a location not covered by the meniscus (Łuczkiewicz et al., 2016) or to a location where the cartilage is much thinner (Si et al., 2021).

It should be mentioned that, even though this research shows a relocation in cartilage loading during graft remodeling, these relocations are only >1 mm for the weakest grafts (e.g., E4, T2, T3, C3, C4, and C5), while during the ligamentization phase of graft remodeling, the graft stiffness restores to around 50%–60% of the native ACL (Janssen and Scheffler, 2014). For graft E1 (68% stiffness) and E2 (37% stiffness), relocations in cartilage peak pressure were <0.8 mm. Nonetheless, these results are calculated during gait, while other functional activities such as stairs walking or squats result in higher contact pressures (van Rossom et al., 2018) and, therefore, will most likely have a greater influence on knee instability.

Interestingly, one of the subjects (subject 2) did not show altered knee kinematics or tibial cartilage pressure patterns, indicating that not every patient might be at risk for the development of OA, which is in line with clinical outcomes (Cheung et al., 2020). It is believed that these patients have a knee that is intrinsically more stable because of knee geometry and that the ACL of these patients endures a relatively small amount of the total stresses and strains in the knee joint, making knee stability not susceptible to changes in graft mechanical properties. This also raises the question if these patients benefit from ACLR and if physical therapy alone would be sufficient.

Of course, this research has limitations. First, only fractions of the forces, moments, and rotations of the stance phase were implemented, and valgus-varus moments were excluded, justified by a discrepancy in the manner of applying the loading conditions in the models compared to the Bergmann data, e.g., the lack of muscle contraction in the models (Bergmann et al., 2014). Next to that, the axial compression for all four models was the same and not adjusted to body weight, however, there seems to be no relation between original subject weight and knee kinematics and cartilage loading found in this research. Moreover, the viscoelasticity of the tendons and ligaments was not included as time dependent loading and responses were not examined. Due to the short time of the stance phase, this is not expected to result in major differences (Wu and Ladin, 1996; Hirokawa and Tsuruno, 2000; Weiss and Gardiner, 2001; Weiss et al., 2005; Peña et al., 2006). However, when introducing multiple gait cycles, or higher-rate or impact activities, viscoelasticity should be included as stress relaxation will lead to increased knee instability (Weiss et al., 2005). In addition, the cartilage mechanical behavior (incompressible Neo-Hookean) is fairly simplified in this study (Henak et al., 2013), but considered to be suitable to investigate differences in local mechanical behavior of the cartilage. Still, the knee range of motion and cartilage loadings found in this study could be an underestimation. Finally, the age of some of the subject can be of influence. Care was taken that the tibial cartilage was, radiographically visible, not damaged, however mechanical properties such as collagen fiber strain and shear stress at the surface could be affected by age. Given that this research focusses on cartilage contact pressure, it is unlikely that these changes influenced the reports results. Nonetheless, the menisci of subject 2 were reported degenerated.

In summary, as the results obtained in this study point in the direction of subject-specificity, this approach should be tested clinically. A retrospective analysis should be performed to assess if ACLR patients with a high intrinsic knee stability, as assessed using the FE analysis presented in this study, result in a low risk for developing OA. In addition, results of this research show that graft remodeling after ACLR can lead to increased knee instability, which suggests the need for novel grafts with mechanical properties that match the native ACL, i.e., anatomical reconstruction of the ACL using ACL allografts (Liu et al., 2023). Alternatively, grafts that possibly circumvent the *in vivo* graft remodeling response, e.g., decellularized grafts, would be an interesting alternative as well (Uquillas et al., 2022). Finally, this model paves the way towards the development of a patient-specific prediction model for knee (in)stability after ACL reconstruction, using, e.g., imaging-based techniques to determine subject-specific material properties (Kang et al., 2016; Wu et al., 2020; Csapo et al., 2021).

## Data Availability

The raw data supporting the conclusion of this article will be made available by the authors, without undue reservation.
