# Supplementary material for: Knee instability caused by altered graft mechanical properties after anterior cruciate ligament reconstruction: the early onset of osteoarthritis?
Source: Front Bioeng Biotechnol. 2023 Aug 24;11:1244954. doi: 10.3389/fbioe.2023.1244954 (PMC10484411; doi:10.3389/fbioe.2023.1244954)
Supplement: Supplementary file 5 [file DataSheet1.docx]

Supplementary Material

Knee instability caused by altered graft mechanical properties after anterior cruciate ligament reconstruction: the early onset of osteoarthritis?

Janne Spierings, Marloes van den Hengel, Rob P. A. Janssen. Bert van Rietbergen, Keita Ito, Jasper Foolen^*^

*** Correspondence:** Jasper Foolen: JFoolen@tue.nl

# Supplementary Figures and Tables

**Supplementary Table 1**. Overview of the material properties. The bulk modulus ($\kappa$) is calculated from D values reported in literature ($\kappa=1/D$). Values in bold are changed in the hypothetical grafts compared to those of the native ACL.

|  | Density (tonnes/mm^3^) | $C_{1}$ (MPa) | $C_{3}$ (MPa) | $C_{4}$ (MPa) | $C_{5}$ (MPa) | $\lambda_{m}$ | $\kappa$ (MPa) | $\lambda_{p}$ |
| --- | --- | --- | --- | --- | --- | --- | --- | --- |
| Cartilage | 1e-9 | 2.54 | - | - | - | - | 100 | - |
| Meniscus | 1e-9 | 4.61 | 0.1197 | 150 | 400 | 1.1019 | 92.16 | - |
| ACL | 1e-9 | 1.95 | 0.0139 | 116.22 | 535.039 | 1.046 | 146.41 | 1.016 |
| PCL | 1e-9 | 3.25 | 0.1196 | 87.178 | 431.063 | 1.035 | 243.9 | 1.0 |
| MCL | 1e-9 | 1.44 | 0.57 | 48.0 | 467.1 | 1.063 | 793.65 | 1.034 |
| LCL | 1e-9 | 1.44 | 0.57 | 48.0 | 467.1 | 1.063 | 793.65 | 1.027 |
| QT | 1e-9 | 2.75 | 0.065 | 115.89 | 777.56 | 1.042 | 206.61 | 1.0 |
| PT | 1e-9 | 2.75 | 0.065 | 115.89 | 777.56 | 1.042 | 206.61 | 1.016 |
| ST | 1e-9 | 2.75 | 0.065 | 115.89 | 512.73 | 1.042 | 206.61 | 1.016 |
| GT | 1e-9 | 2.75 | 0.065 | 115.89 | 791.4 | 1.042 | 206.61 | 1.016 |
| E1 | 1e-9 | 1.95 | 0.0139 | **110** | **310** | 1.046 | 146.41 | 1.016 |
| E2 | 1e-9 | 1.95 | 0.0139 | **100** | **150** | 1.046 | 146.41 | 1.016 |
| E3 | 1e-9 | 1.95 | 0.0139 | **90** | **90** | 1.046 | 146.41 | 1.016 |
| E4 | 1e-9 | 1.95 | 0.0139 | **25** | **25** | 1.046 | 146.41 | 1.016 |
| T1 | 1e-9 | 1.95 | **0.001** | 116.22 | 535.039 | **1.065** | 146.41 | 1.016 |
| T2 | 1e-9 | 1.95 | **5e-5** | 116.22 | 535.039 | **1.09** | 146.41 | 1.016 |
| T3 | 1e-9 | 1.95 | **5e-7** | 116.22 | 535.039 | **1.13** | 146.41 | 1.016 |
| C1 | 1e-9 | 1.95 | **0.005** | **110** | **310** | **1.06** | 146.41 | 1.016 |
| C2 | 1e-9 | 1.95 | **0.001** | **110** | **200** | **1.07** | 146.41 | 1.016 |
| C3 | 1e-9 | 1.95 | **5e-5** | **90** | **90** | **1.075** | 146.41 | 1.016 |
| C4 | 1e-9 | 1.95 | **5e-5** | **50** | **50** | **1.09** | 146.41 | 1.016 |
| C5 | 1e-9 | 1.95 | **5e-7** | **25** | **25** | **1.13** | 146.41 | 1.016 |
| DEF | 1e-9 | **0.1** | **5e-15** | **0.01** | **0.01** | **1.6** | 146.41 | 1.016 |


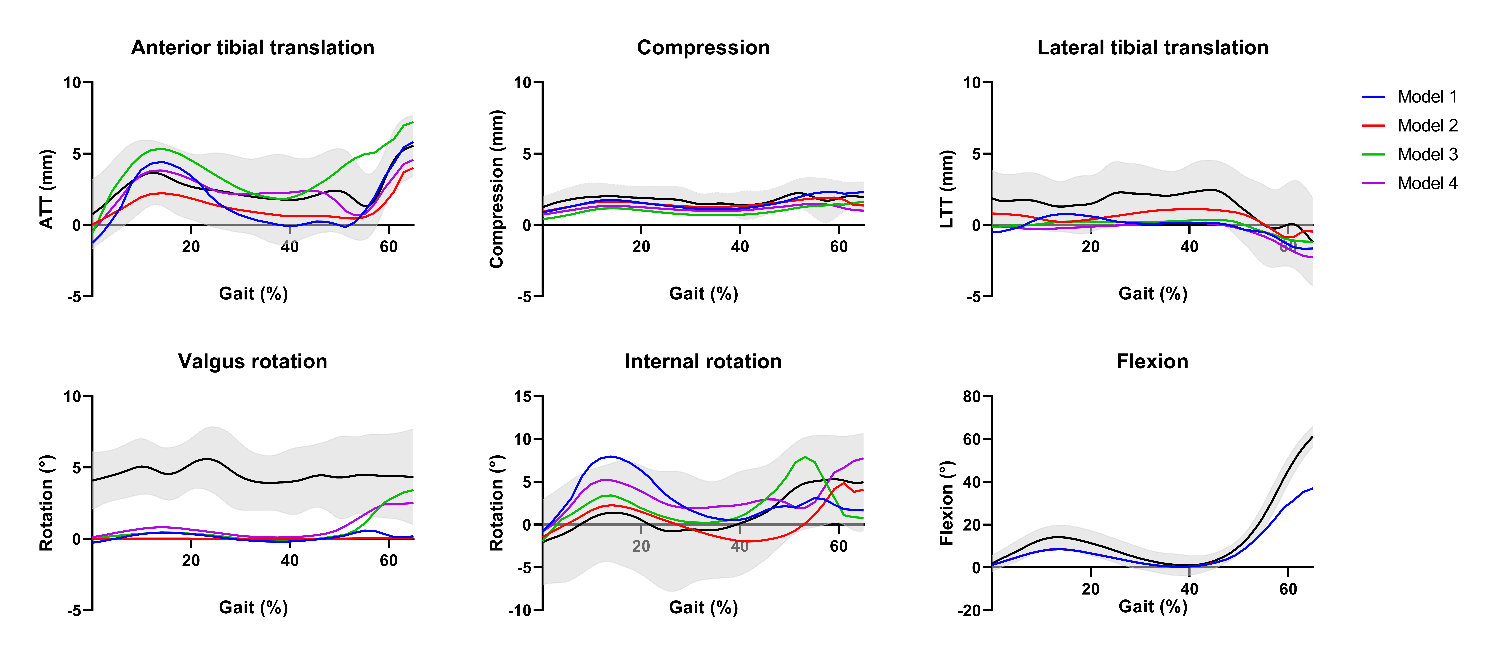


**Supplementary Figure 1.** **Fractions of force, momentum, and flexion were adjusted to obtain translations and rotations similar to the *in vivo* findings by Gray *et al*.** (Gray et al., 2019)**.** ATT, compression, lateral tibial translation (LTT), valgus rotation, IR, and flexion of all models and the average values found by Gray *et al*. in black, with standard deviation in grey. To obtain these results 50% force, 10% moment, and 60% flexion was applied.


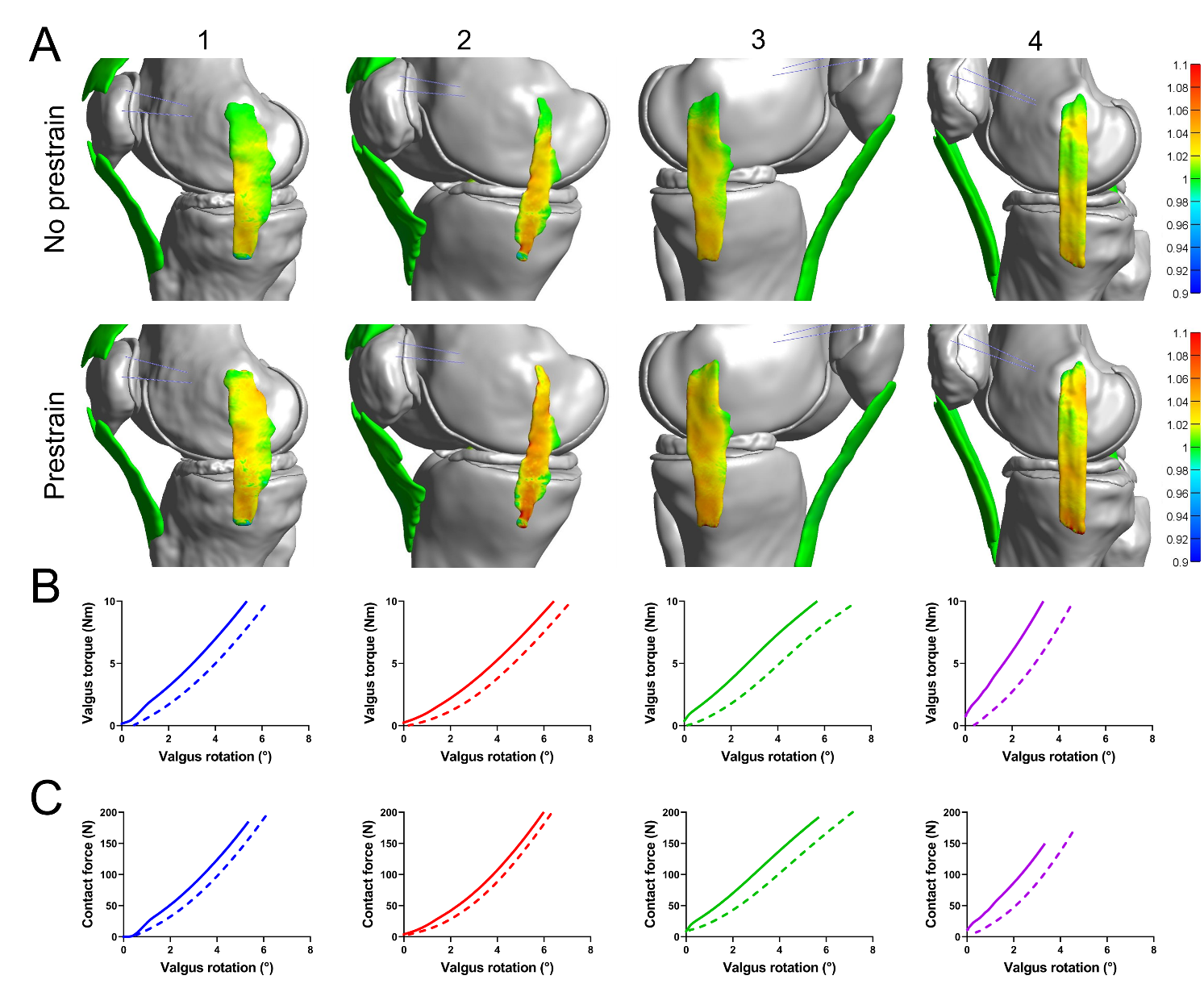


**Supplementary Figure 2.** **Adding pre-strain to the tendons and ligaments results in increased knee stability. A)** Pre-strain stretch in the MCL at 3° valgus rotation both with and without applied pre-strain. The pre-strain stretch is the sum of the stretch induced by the *in situ* fiber stretch and the stretch as a result of valgus rotation. **B)** Valgus rotation over valgus torque for all models with (full line) and without (dashed line) applied pre-strain. **C)** Contact force on the lateral condyle per degree of valgus rotation with (full line) and without (dashed line) applied pre-strain.


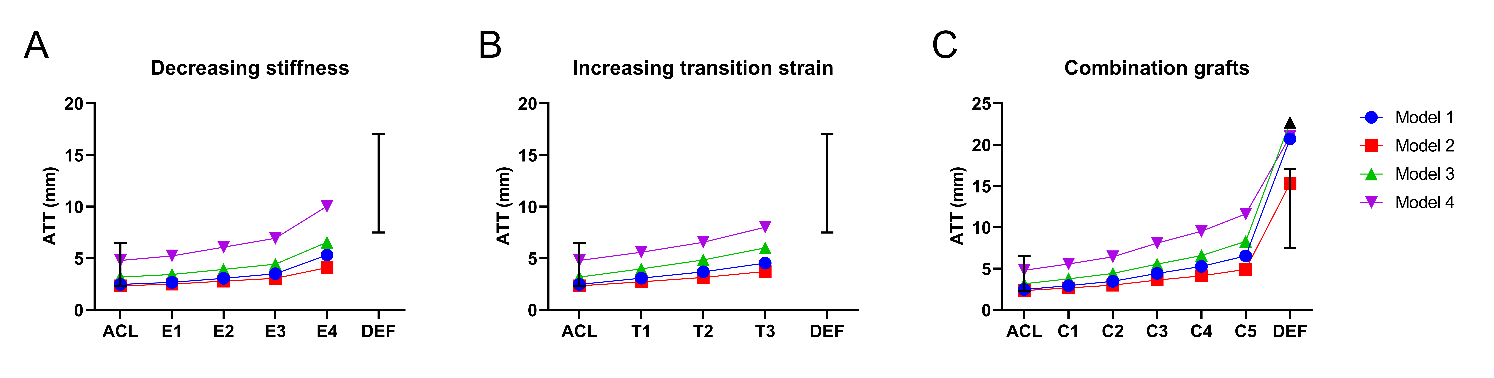


**Supplementary Figure 3. Decreasing graft stiffness and/or increasing graft laxity results in an increased ATT.** ATT as a result of 134 N applied force for **A)** grafts with a decreasing stiffness, **B)** grafts with an increasing transition strain, and **C)** combination grafts. The black range indicates experimental values obtained from literature (Drews et al., 2017; Höher et al., 2001; Kim et al., 2015; Yagi et al., 2002; Zantop et al., 2007). For ACL DEF in model 3 (black triangle) the augmented Lagrangian method rather than a penalty method was used to solve all contacts involving the ACL to overcome numerical instability.


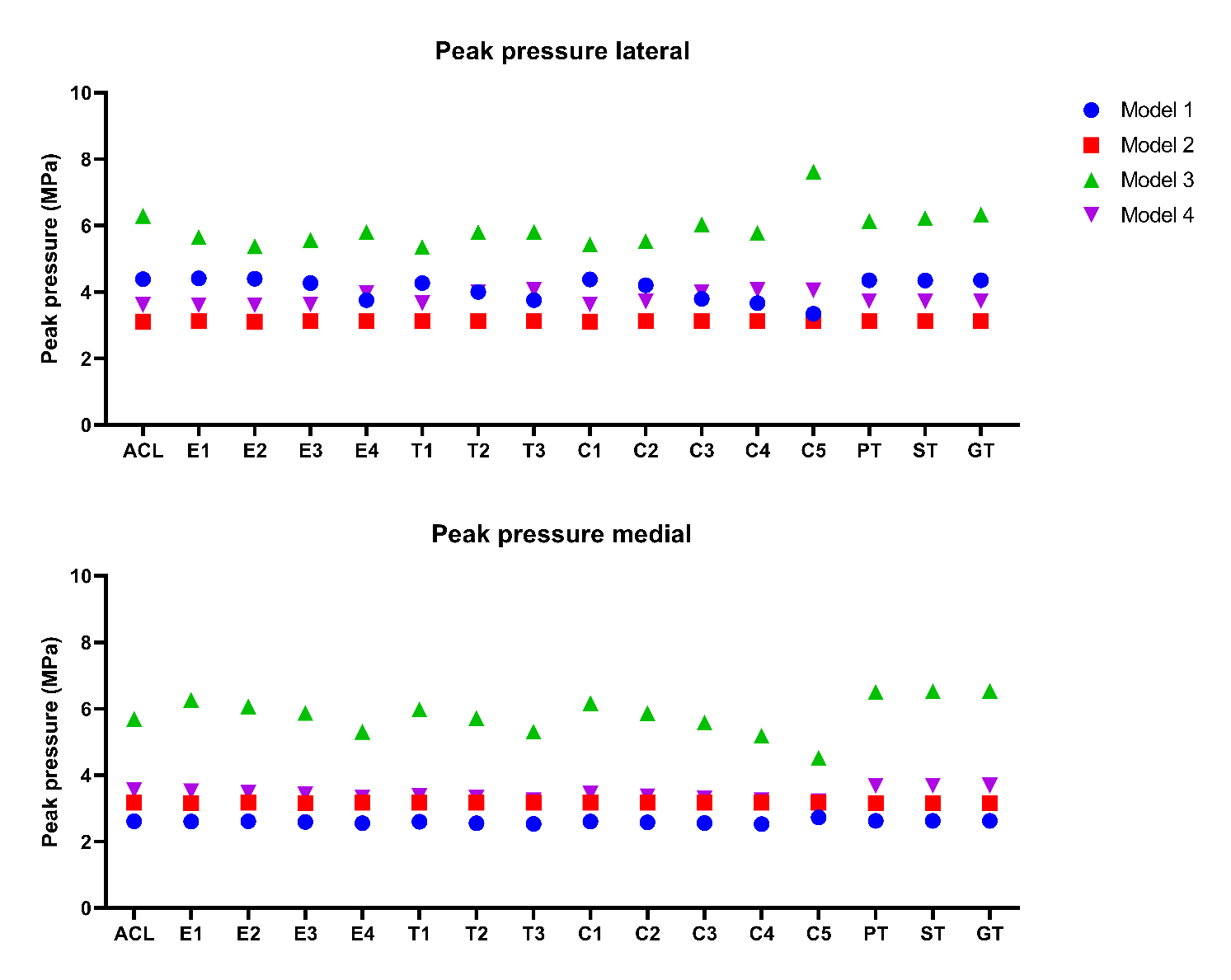


**Supplementary Figure 4. Peak pressure is not significantly different with respect to the native ACL for all models.** The contact pressure was recorded at all nodes of the tibial cartilage and the maximum was selected.


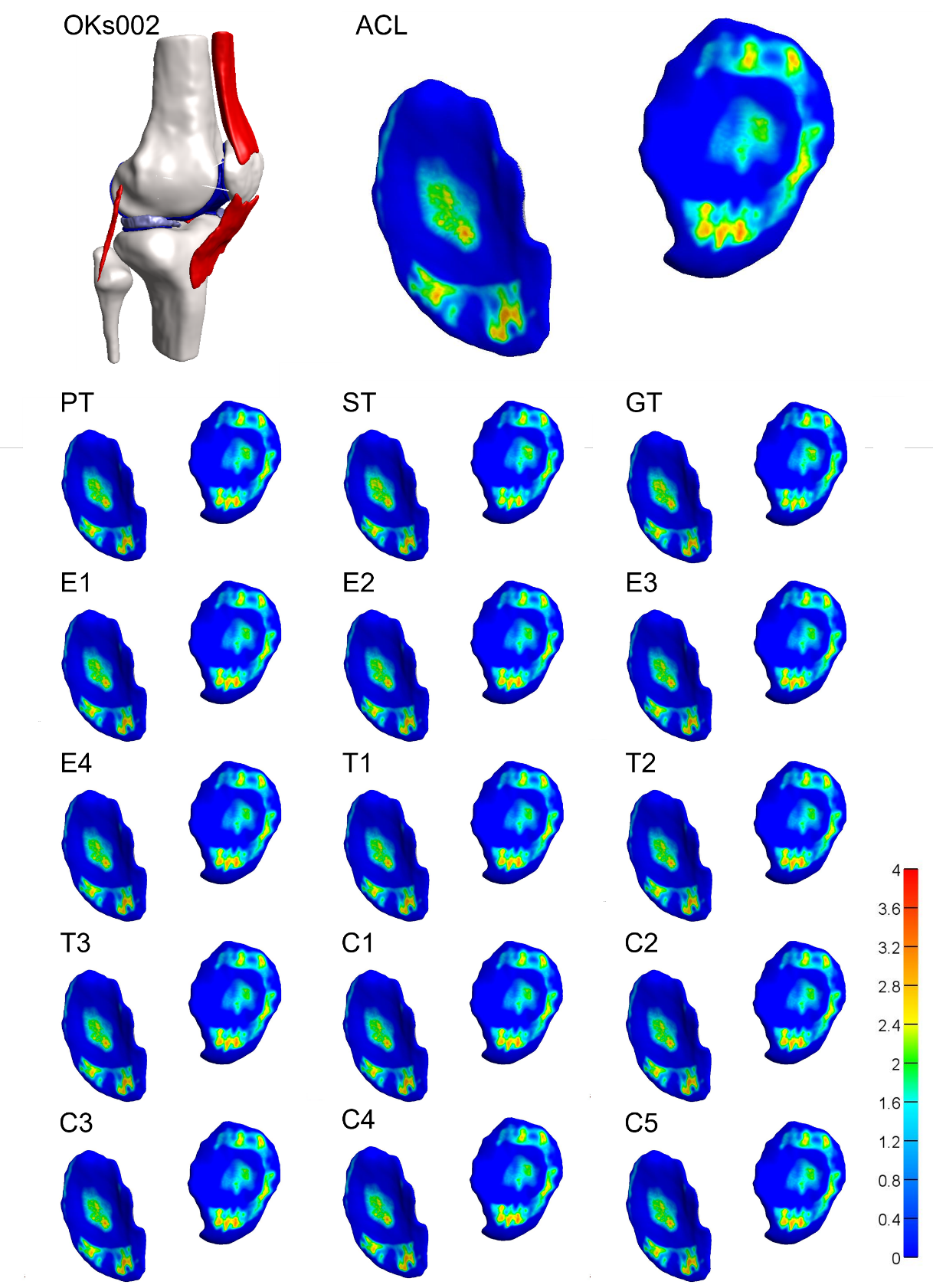


**Supplementary Figure 5. Altering graft mechanical properties results in a relocation of tibial cartilage contact pressure [MPa].** Visual representation of the tibial cartilage contact pressure distribution of model 2 for the native ACL, the tendon grafts (PT, ST, and GT), grafts with a decreasing stiffness (E1-4), grafts with an increasing transition strain (T1-3), and a combination of both (C1-5). Left: medial tibial cartilage; right: lateral tibial cartilage.


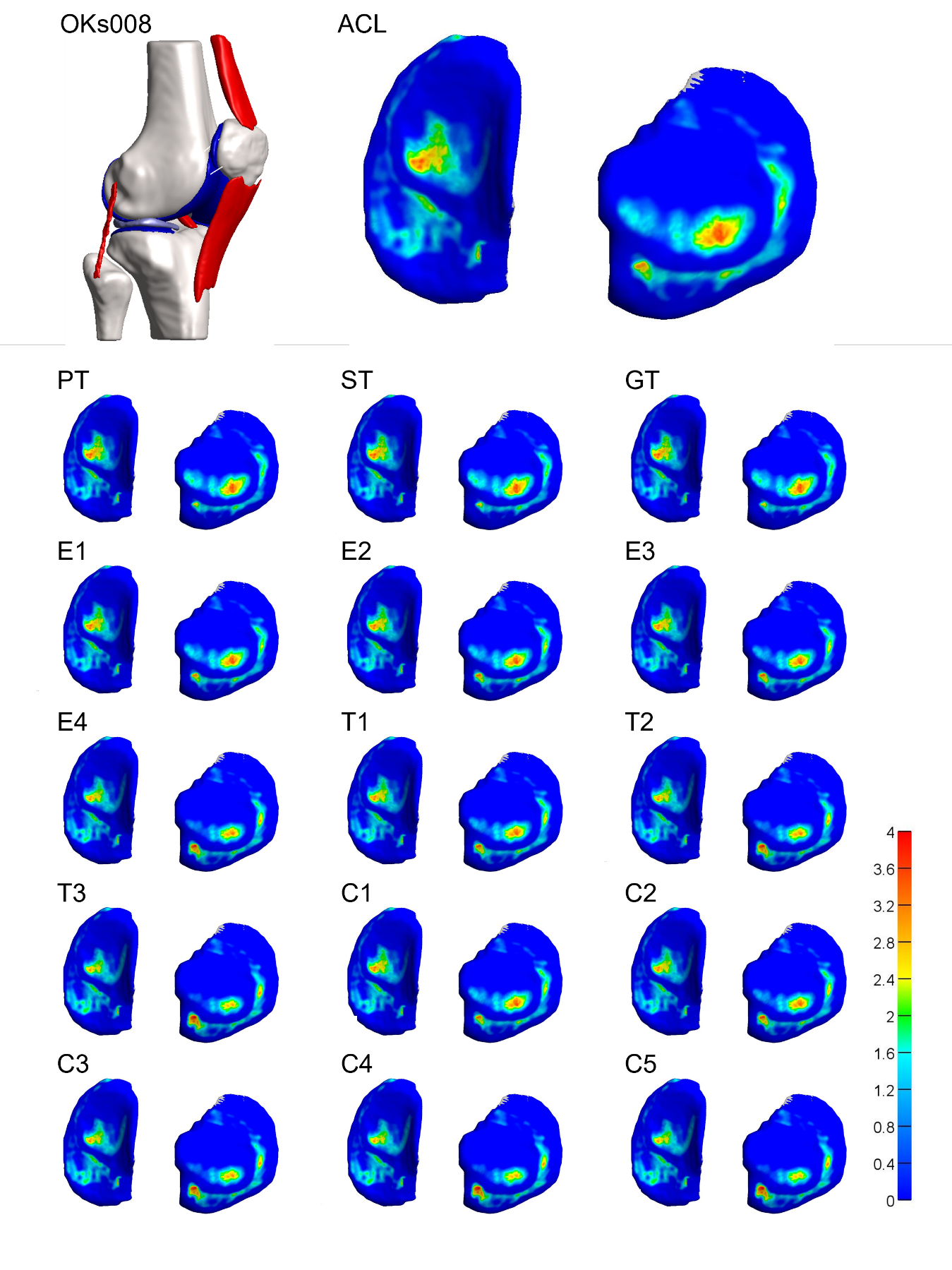


**Supplementary Figure 6. Altering graft mechanical properties results in a relocation of tibial cartilage contact pressure [MPa].** Visual representation of the tibial cartilage contact pressure distribution of model 4 for the native ACL, the tendon grafts (PT, ST, and GT), grafts with a decreasing stiffness (E1-4), grafts with an increasing transition strain (T1-3), and a combination of both (C1-5). Left: medial tibial cartilage; right: lateral tibial cartilage.
